# Optimization of Fermentation and Transcriptomic Analysis of a High-Protein-Producing *Galactomyces geotrichum* Strain

**DOI:** 10.3390/foods15111996

**Published:** 2026-06-03

**Authors:** Lu Sun, Na Zhang, Wei Hua, Yanling Cheng, Shengquan Mi, Cheng Zhou

**Affiliations:** 1 College of Biochemical Engineering, Beijing Union University, Beijing 100023, China; 18856160146@163.com (L.S.); 20237003@buu.edu.cn (N.Z.); huawei0917@outlook.com (W.H.); cheng1012cn@aliyun.com (Y.C.); 2 Beijing Key Laboratory for Utilization of Biomass Wastes, Beijing 100023, China

**Keywords:** *Galactomyces geotrichum*, protein yield, submerged fermentation, functional gene dissection

## Abstract

The global shortage of protein resources drives the need for sustainable microbial protein production. *Galactomyces geotrichum* can convert various substrates into high-quality single-cell protein (SCP). In this study, a strain of *Galactomyces geotrichum* (Gg6) was isolated and identified from cheese. Through single-factor tests and orthogonal array design, optimal fermentation conditions were established, achieving a protein content of 54.56 ± 0.05% and a protein yield of 6.12 ± 0.11 g/L in shake flask cultivation (*n* = 3). Phosphate (PO_4_^3−^) was identified as the key regulatory ion, and supplementation with potassium dihydrogen phosphate (KH_2_PO_4_) significantly enhanced amino acid accumulation. Transcriptomic analysis revealed that KH_2_PO_4_ reprograms central carbon metabolism to support amino acid biosynthesis. These findings provide an experimental and theoretical basis for the industrial application and genetic improvement of this strain in microbial protein production.

## 1. Introduction

With the continuous growth of the global population, the limitations of traditional animal and plant protein resources have become increasingly prominent, making the search for efficient and sustainable protein production methods a critical research priority [1]. The emergence of alternative proteins (including microbial protein, insect protein, cultured meat, and plant-based meat substitutes) is expected to better meet global protein demand by transforming the food protein manufacturing model from traditional farming and cultivation to workshop-based production [2]. Among these, microbial protein (also known as single-cell protein, SCP) has become one of the most promising alternative proteins due to its advantages of high production efficiency, high digestibility, and environmental sustainability [3]. SCP is derived from microalgae, fungi, yeast, and bacteria [4]. It not only contains abundant protein but also comprises amino acids, carbohydrates, lipids, vitamins, and minerals, making it a nutritious alternative to animal or plant proteins [5]. Currently, SCP has been successfully applied in various fields, including as a protein source in fish meal, foam stabilizers, and potential packaging materials [6,7,8], and has shown promising prospects in animal feed applications [9,10].

*Galactomyces geotrichum* (the sexual form of *Geotrichum candidum*) is widely found in soil, water, air, and dairy products [11]. It can utilize residual sugars, organic acids, proteins, and fatty acids from organic waste, converting them into protein-rich biomass that is easily absorbed by animals [12]. Studies have shown that *Galactomyces geotrichum* can be used to produce high-quality microbial protein feed from apple pomace [13]. Moreover, mixed culture fermentation with *Candida utilis* significantly increases protein content, achieving an essential amino acid profile comparable to that of commercial protein [14]. Therefore, it is necessary to achieve a high protein yield (relative to previously reported levels) of *Galactomyces geotrichum* through fermentation optimization and to explore the metabolic pathways involved in its protein synthesis.

In this study, a *Galactomyces geotrichum* strain (the sexual form of *Geotrichum candidum*) was isolated from cheese samples and identified through morphological observation and ITS sequencing [15]. A systematic optimization strategy combining single-factor experiments and orthogonal array design was employed to optimize fermentation conditions [16,17]. Protein content and biomass were determined using the Kjeldahl method and dry weight measurement, respectively [18,19]. To investigate how KH_2_PO_4_ promotes protein accumulation, we examined its ionic effects, analyzed the amino acid profile, and performed transcriptomic analysis to identify enriched metabolic pathways. This study provides a theoretical basis for the industrial application and genetic improvement of *G. geotrichum*.

## 2. Materials and Methods

### 2.1. Strain Screening and Preservation

In an ultra-clean workbench, a piece of President brand cheese (237 Andingmen Inner Street, Dongcheng District, Beijing, China) was aseptically cut and placed on a potato dextrose agar (PDA) (Solarbio, Beijing, China) plate. The plate was incubated at 28 °C for 24–48 h, and plates with vigorous microbial growth were selected for subsequent isolation. White colonies were picked and inoculated into potato dextrose broth (PDB) medium (Solarbio, Beijing, China), followed by incubation at 28 °C with shaking at 180 rpm for 24–48 h to obtain a cell suspension. A plate cell count on PDA was then performed. Plates were incubated at 28 °C for 24–48 h. Colony morphological characteristics were observed periodically. Colonies morphologically resembling *Geotrichum candidum* were selected and subjected to continuous streak purification on plates. The purified strain was mixed with sterile 60% glycerol (1:1, *v*/*v*) and stored at −80 °C.

### 2.2. Identification of Screened Strains

The purified strains obtained from isolation were inoculated onto PDA solid medium and incubated at 28 °C for 3–5 days. Colony morphology and color were observed. Subsequently, mycelia and spores were picked from the solid medium, stained with Lactophenol Cotton Blue, and examined under a Leica DM2500 light microscope (Leica Microsystems, Wetzlar, Germany) for morphological characteristics of hyphae and spores. Total DNA of the two screened strains was extracted using a DNA extraction kit (Solarbio, Beijing, China). The extracted DNA was subjected to purity verification and then amplified by PCR. The internal transcribed spacer (ITS) region was amplified using primers ITS1 (5′-TCCGTAGGTGAACCTGCGG-3′) and ITS4 (5′-TCCTCCGCTTATTGATATGC-3′). PCR products of approximately 200 bp were sent to Beijing Tsingke Biotechnology Co., Ltd. for paired-end sequencing. The obtained sequences were compared against the NCBI database using BLAST (https://blast.ncbi.nlm.nih.gov/Blast.cgi) for sequence alignment and homology search. Finally, a phylogenetic tree was constructed using MEGA11.0 software [20].

### 2.3. Determination of Growth Curve

The growth curve was determined by the dry weight method as described in [18]. A loopful of *Geotrichum candidum* preserved in a cryotube at −80 °C was taken and spread onto a PDA plate, followed by incubation at 28 °C for 24 h. A loopful of the culture was then streaked for purification and incubated at 28 °C for another 24 h. Subsequently, a loopful of the purified culture was inoculated into 100 mL of PDB liquid medium (Solarbio, Beijing, China) and incubated at 28 °C with shaking at 180 rpm for 24 h to prepare the seed culture. The seed culture was inoculated into 100 mL of PDB liquid medium at a 1% (*v*/*v*) inoculum size. The quantitative filter paper was dried to constant weight, and its weight was recorded. The mycelia were collected by repeated vacuum filtration with distilled water to remove the culture medium. The collected mycelia were directly placed in an electrothermal constant-temperature drying oven (Yiheng, Shanghai, China) and dried at 80 °C to constant weight. A growth curve was then plotted based on the dry weight measurements (*n* = 3).

### 2.4. Optimization of PDB Inorganic Salt Supplementation

Inorganic salt ions are known to be essential during microbial cultivation. Therefore, the effects of supplementing the PDB medium with different concentrations of KCl and KH_2_PO_4_ (0, 1.0, 1.5, 2.0, and 2.5 g/L) as well as ZnSO_4_ (0, 0.02, 0.03, 0.04, and 0.05 g/L) on the protein content and yield of *Galactomyces geotrichum* strain 6 were investigated. A loopful of *Geotrichum candidum* preserved in a cryotube at −80 °C was taken and spread onto a PDA plate and incubated at 28 °C for 24 h. A loopful of the culture was then streaked for purification and incubated at 28 °C for another 24 h. Subsequently, a loopful of the purified culture was inoculated into 100 mL of PDB liquid medium and incubated at 28 °C with shaking at 180 rpm for 24 h to prepare the seed culture. The seed culture was inoculated at 5% (*v*/*v*) into 100 mL of fermentation medium and incubated at 28 °C with shaking at 180 rpm for 72 h. According to the Kjeldahl method [19], approximately 0.2 g of ground lyophilized mycelial powder was digested with 10 mL of concentrated sulfuric acid in the presence of a catalyst (copper sulfate and potassium sulfate). The released ammonia was then distilled and titrated with 0.1 M hydrochloric acid. The total nitrogen content was calculated and multiplied by a conversion factor of 6.25 to obtain the crude protein content (%). The protein yield (g/L) was calculated as the product of biomass (g/L) and protein content (%). This experiment was conducted to provide a reference for the subsequent optimization of inorganic salt supplementation in the culture medium.

### 2.5. Fermentation Process Optimization

Due to the high cost of commercial PDB medium, we re-screened low-cost medium components that could improve the protein yield of the strain. The basal fermentation medium consisted of potato extract 5 g/L (Huasheng, Tianjin, China), NaCl 5 g/L (Haohong, Shanghai, China), glucose 15 g/L (Solarbio, Beijing, China), and peptone (Solarbio, Beijing, China). Through single-factor experiments, xylose (Yuanye, Shanghai, China), glycerol (Solarbio, Beijing, China), and fructose (Solarbio, Beijing, China) were used to replace glucose in the fermentation medium as carbon sources for screening, and concentration gradients of 10, 20, 40, 60, and 80 g/L were set for optimization of the optimal carbon source. For nitrogen source screening, yeast extract powder (Aoboxing, Beijing China), fish peptone (Huasheng, Tianjin, China), and soybean peptone (Solarbio, Beijing, China) were used to replace peptone (Solarbio, Beijing, China) in the medium, and concentration gradients of 5, 10, 15, 20, and 25 g/L were set for optimization of the optimal nitrogen source. Additionally, referring to the strategy of supplementing inorganic salt ions in PDB medium, and considering that magnesium ion serves as a cofactor for various key metabolic enzymes (e.g., kinases and polymerases) and participates in ATP binding and protein synthesis, the present study further investigated the effects of different concentrations of inorganic salts, including KH_2_PO_4_ (0, 0.5, 1, 1.5, and 2 g/L), MgSO_4_ (0, 0.5, 1, 1.5, and 2 g/L), and ZnSO_4_ (0, 0.01, 0.02, and 0.03 g/L). Furthermore, fermentation conditions including inoculum size (1%, 3%, 5%, and 7%), temperature (24 °C, 28 °C, and 32 °C), and pH (4, 6, 8, and 10) were investigated to evaluate their effects on strain growth and protein accumulation, and the factors with significant effects were determined. All fermentation optimization experiments were performed in triplicate.

### 2.6. Orthogonal Test Optimization

Based on the results of single-factor experiments, four factors that significantly influenced protein yield were selected: glucose concentration (A), yeast extract powder concentration (B), ZnSO_4_ concentration (C), and pH (D). The experimental factors and levels are shown in Table 1, and the orthogonal test design is shown in Table 2. Glucose concentration (A), yeast extract powder concentration (B), ZnSO_4_ concentration (C), and pH (D) were investigated as factors to evaluate their effects on the growth and protein yield of *G. geotrichum* 6. The orthogonal test was conducted in triplicate, and the results were expressed as mean ± standard deviation (SD).

### 2.7. Transcriptomic Analysis Based on Potassium Dihydrogen Phosphate Metabolism

To elucidate the underlying mechanism by which KH_2_PO_4_ promotes strain growth and protein synthesis, this study systematically investigated three aspects: ion effect analysis, amino acid composition analysis, and transcriptomic analysis. First, to identify the key ionic component of KH_2_PO_4_, the effects of K^+^ and PO_4_^3−^ on biomass and protein content were examined. This was achieved by setting up treatment groups with KCl (providing an equivalent molar concentration of K^+^) and NaH_2_PO_4_ (providing an equivalent molar concentration of PO_4_^3−^), using 1 g/L KH_2_PO_4_ as the reference and PDB medium without inorganic salt supplementation as the blank control.

On this basis, cells cultured in basal PDB medium and KH_2_PO_4_-supplemented PDB medium for 72 h were collected to determine the contents of 18 free amino acids. Lyophilized mycelial powder (0.05–0.1 g) was extracted with 1 mL of distilled water at 50 °C for 30 min. After centrifugation (10,000 rpm, 5 min), 1 mL of supernatant was mixed with 1 mL of 10% TCA and allowed to stand at room temperature for 30 min. The mixture was centrifuged (10,000 rpm, 5 min, 4 °C), and the supernatant was filtered (0.22 μm). The 17 free amino acids (excluding tryptophan) were determined using an automatic amino acid analyzer (Sykam, Beijing, China). For tryptophan, lyophilized mycelial powder (0.05 g) was extracted with 2 mL of distilled water at 50 °C for 30 min with shaking. After centrifugation (10,000 rpm, 5 min), the supernatant was filtered (0.22 μm) and analyzed by fluorescence spectrophotometry (Ex 280 nm, Em 360 nm) following standard protocols. Tryptophan content was quantified using an external standard curve. The content of each free amino acid was expressed as a percentage of the total free amino acid content.

Additionally, transcriptome analysis was performed. The strain was inoculated separately into basal PDB medium and PDB medium supplemented with 1 g/L KH_2_PO_4_, and cultured at 28 °C with shaking at 180 rpm for 72 h. Subsequently, the cells were collected and treated with liquid nitrogen. Three biological replicates were set for each group (n = 3). Total RNA was extracted, and RNA integrity was verified by Tsingke Biotech (Beijing, China) following the company’s standard protocol. Sequencing was performed on an Illumina platform (PE150). Clean reads were aligned to the reference genome using HISAT2 (v2.2.1). Gene expression was quantified as FPKM using StringTie (v2.0.4). Differential expression analysis was performed using DESeq2 (v1.26.0), with significance defined as |log_2_FC| ≥ 1 and FDR (*p*adj) < 0.05 (Benjamini–Hochberg correction).

### 2.8. Statistical Analysis

Statistical analysis of the data was performed using SPSS 27.0 software. Comparisons among multiple groups were conducted by one-way analysis of variance (ANOVA). For multiple comparisons, different lowercase letters (a, b, c, etc.) indicate significant differences between groups (*p* < 0.05). In the figures, the same letters indicate no significant difference between groups, whereas different letters indicate significant differences.

## 3. Results

### 3.1. Identification and Physiological Characteristics Analysis of the Strain

**Figure 1:**
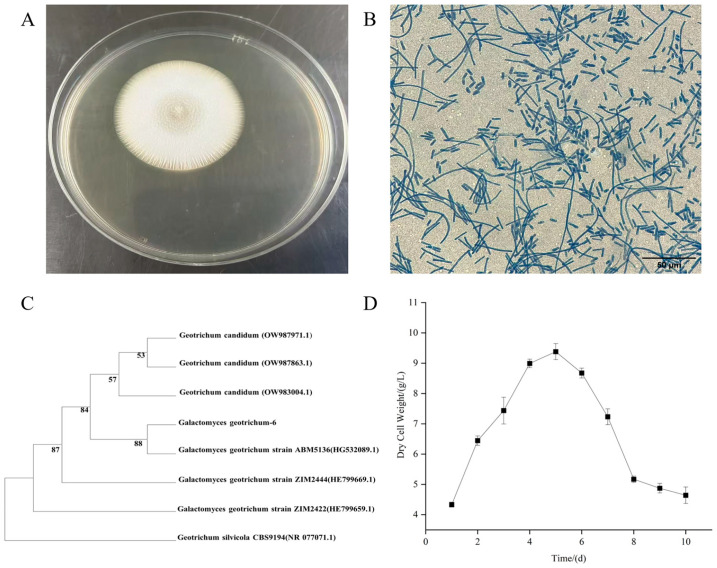
Isolation, identification and growth characterization of *G. geotrichum* strain Gg6. (**A**) Colony morphology. (**B**) Microscopic observation. (**C**) Phylogenetic tree based on ITS gene sequences. (**D**) Growth curve; data are presented as mean ± SD (*n* = 3).

A strain exhibiting unique growth characteristics on PDA solid medium was isolated from President brand cheese samples through screening and separation. The strain formed white, circular colonies (Figure 1A) and was temporarily named strain No. 6 for subsequent studies. After staining with lactophenol cotton blue and observation under a microscope (Figure 1B), the strain displayed slender, extensively branched hyphae with sporulation, and the spores were long and cylindrical in shape. Based on comparative analysis with the “Fungal Identification Manual” [21], the strain was preliminarily identified as *G. candidum*. To further confirm its taxonomic status, the sequencing results of the strain were compared with the BLAST sequence library in the NCBI database, and a phylogenetic tree was constructed accordingly. Analysis of the phylogenetic tree (Figure 1C) revealed that strain No. 6 clustered with *Galactomyces geotrichum* (the sexual form of *G. candidum*) on the same node, indicating a very close genetic evolutionary relationship. This confirmed that strain No. 6 was *Geotrichum candidum*, and it was named *G. geotrichum* 6 (hereinafter referred to as Gg6). The sequence data have been deposited in the NCBI GenBank database under accession number PZ359123.

To determine the subsequent fermentation time for this strain, its growth curve was measured (Figure 1D). The results showed that within 0–5 days, the biomass dry weight continued to increase rapidly. After reaching the peak of growth on day 5 or 6, it directly entered the decline phase, with the dry weight gradually decreasing. The overall trend showed a pattern of “rapid proliferation—direct decline after the peak,” with no obvious stationary phase observed. This indicated that after reaching maximum biomass accumulation on day 5 or 6, cells inside the mycelial pellets likely underwent autolysis mainly due to rapid nutrient depletion and accumulation of metabolic products, as they could not access nutrients, leading to an overall decrease in biomass. This characteristic often results in a very brief stationary phase in the growth curve of the filamentous fungus *G. candidum*, which may even transition directly from the logarithmic phase to the decline phase. which is consistent with previous reports [22]. Considering the growth characteristics of Gg6, 72 h was selected as the culture time for subsequent fermentation experiments.

### 3.2. Results of Optimization of PDB Inorganic Salt Supplementation

**Figure 2:**
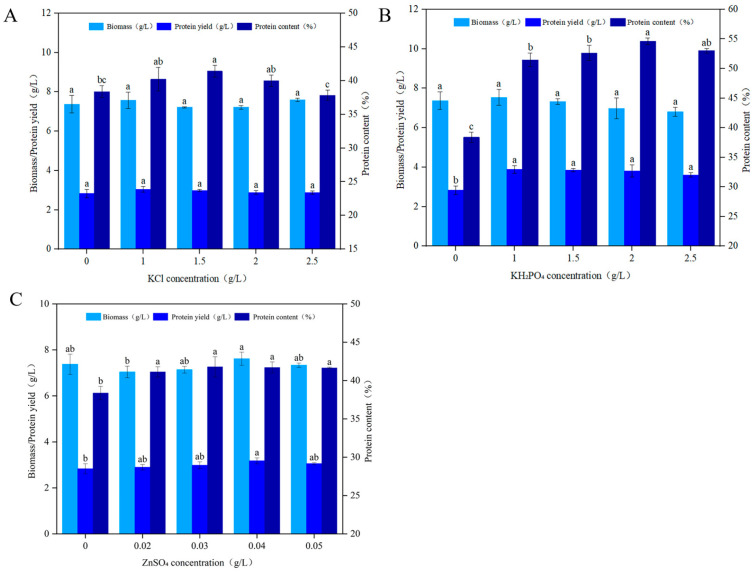
Optimization results of exogenous inorganic salts. (**A**) Optimization results of KCl concentration. (**B**) Optimization results of KH_2_PO_4_ concentration. (**C**) Optimization results of ZnSO_4_. Data are presented as mean ± SD *(n* = 3). Bars bearing different lowercase letters (e.g., a, b, c) differ significantly (*p* < 0.05), whereas bars sharing the same letter are not significantly different.

This study further investigated the effects of adding different concentrations of KCl (0, 1.0, 1.5, 2.0, 2.5 g/L) to the basal PDB medium on strain growth and protein accumulation. The results are shown in Figure 2A. Without KCl addition, the biomass was 7.4 g/L; when the KCl concentration was 1.0 g/L, the biomass increased to the highest value of 7.6 g/L; at concentrations of 1.5 g/L and 2.0 g/L, the biomass slightly decreased to 7.2 g/L; at a concentration of 2.5 g/L, the biomass was 7.6 g/L. The overall fluctuation was small, indicating that KCl had no significant effect on cell growth. Without KCl addition, the protein content was 38.0%; after adding KCl, although the protein content first increased and then decreased with increasing concentration, the increase was not significant. Therefore, based on the significance analysis of protein yield, the addition of inorganic salt ions like KCl to the basal medium had no significant effect on the protein yield of the strain.

Figure 2B shows the results of the effect of adding different concentrations of inorganic salt ions like KH_2_PO_4_ on the growth and protein accumulation of strain Gg6. The results indicated that the addition of inorganic salt ions had no significant effect on biomass. However, after adding inorganic salt ions like KH_2_PO_4_, the protein content increased significantly. At a concentration of 2.0 g/L, the protein content reached a peak of 54%, which was 16% higher than that of the original basal medium. Therefore, the addition of KH_2_PO_4_ was considered a beneficial factor under the initial basal medium conditions and served as a reference for the selection of inorganic salts during the subsequent fermentation optimization process.

Figure 2C shows the results of the effect of adding different concentrations of inorganic salt ions such as ZnSO_4_ on the growth and protein accumulation of strain Gg6. In the control group without ZnSO_4_ addition, the biomass was 7.3 g/L. When the ZnSO_4_ concentration increased to 0.02 g/L, the biomass slightly decreased; when the concentration reached 0.04 g/L, the biomass reached the maximum value within the experimental range (7.6 g/L); when the concentration was further increased to 0.05 g/L, the biomass was comparable to that of the control group. The results showed that within the ZnSO_4_ concentration range of 0.02–0.05 g/L, the inorganic salt ions ZnSO_4_ did not significantly inhibit strain growth, and at 0.04 g/L, it had a certain promoting effect on biomass. The protein content remained at approximately 41%, with no significant changes. Although the protein yield remained relatively stable across all ZnSO_4_ treatment groups, based on significance analysis, a significant increase in protein yield was observed at 0.04 g/L compared to the control group. Therefore, the addition of ZnSO_4_ was considered a beneficial factor and served as a reference for inorganic salt selection during the subsequent fermentation optimization process.

### 3.3. Optimization of Fermentation Process Results

#### 3.3.1. Carbon Source Optimization

**Figure 3:**
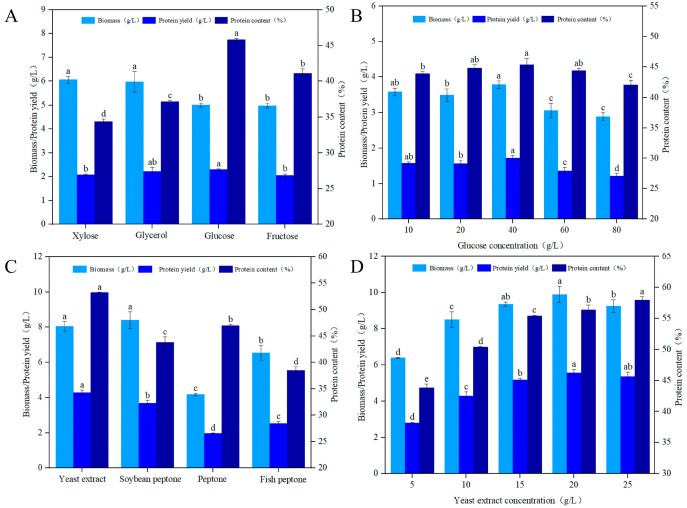
Results of carbon and nitrogen source optimization. (**A**) Effects of different carbon sources on fermentation of Gg6. (**B**) Effects of different glucose concentrations on fermentation of Gg6. (**C**) Effects of different nitrogen sources on fermentation of Gg6. (**D**) Effect of yeast extract concentration on fermentation of Gg6. Data are presented as mean ± SD (*n* = 3). Bars bearing different lowercase letters (e.g., a, b, c) differ significantly (*p* < 0.05), whereas bars sharing the same letter are not significantly different.

As shown in Figure 3A, single carbon sources (xylose, glucose, glycerol, fructose) were selected to investigate their effects on the growth and protein accumulation of strain Gg6. The results showed that different carbon sources had significant effects on cell growth and protein expression. In terms of biomass accumulation, xylose as the carbon source yielded the highest biomass, reaching 6 g/L, which was slightly higher than that of glycerol and significantly higher than that of glucose and fructose. This indicates that xylose as a single carbon source is most favorable for cell proliferation and growth. In terms of protein synthesis, both glycerol and glucose exhibited promoting effects, with protein yields reaching 2.2 g/L, which were higher than those of xylose and fructose. Further analysis of protein content revealed that the glucose had the highest protein content, followed by the glycerol, whereas the xylose, despite having the highest biomass, exhibited the lowest protein content among all carbon sources. This result indicates that although xylose promotes cell growth, it is not conducive to protein accumulation; in contrast, glucose and glycerol, while maintaining moderate cell growth, more effectively promote protein synthesis. Considering economic factors, glucose was selected as the optimal carbon source for subsequent optimization.

As shown in Figure 3B, the experimental results demonstrated that glucose concentration significantly affected both cell growth and protein yield. In terms of biomass accumulation, the biomass reached its maximum value of 3.7 g/L when the glucose concentration was 40 g/L; when the concentration was increased to 60 g/L and 80 g/L, the biomass decreased, showing a clear downward trend. This suggests that excessively high glucose concentrations may have an inhibitory effect on cell growth. As the glucose concentration increased, both protein yield and protein content initially increased and then decreased. When the glucose concentration was 40 g/L, both protein yield and protein content reached their peak values; when the concentration was increased to 60 g/L and 80 g/L, both protein yield and protein content decreased. This trend further confirms the inhibitory effect of high glucose concentrations on protein synthesis.

#### 3.3.2. Nitrogen Source Optimization

This study investigated the effects of four different nitrogen sources—yeast extract, soybean peptone, peptone, and fish peptone—on Gg6 fermentation. The results are shown in Figure 3C. The experimental results indicated that different nitrogen sources had significant effects on cell growth and protein expression. Among them, soybean peptone as the nitrogen source yielded the highest biomass, with the yeast extract being slightly lower, but both were significantly higher than the fish peptone and peptone. This indicates that soybean peptone as a nitrogen source is most favorable for cell proliferation and growth. In terms of protein yield, yeast extract exhibited the best promoting effect, reaching a value as high as 4.3 g/L, which was significantly higher than those of the soybean peptone, fish peptone, and peptone. Further analysis of protein content revealed that the yeast extract had the highest protein content, reaching 53%, followed by the peptone, whereas the soybean peptone, which had the highest biomass, had a protein content of only 43%. Notably, although soybean peptone promoted biomass accumulation, its protein yield was lower than that of the yeast extract; in contrast, when yeast extract was used as the nitrogen source, it achieved a higher protein content while maintaining relatively high biomass. This result indicates that yeast extract is more favorable for promoting protein synthesis. Considering all factors comprehensively, yeast extract performed best in terms of both protein yield and protein content, and its biomass was second only to that of soybean peptone. Therefore, yeast extract was selected as the optimal nitrogen source for subsequent optimization.

The effect of different yeast extract concentrations on Gg6 fermentation was further investigated. The results are shown in Figure 3D. The experimental results indicated that the biomass of the strain initially increased and then decreased with increasing yeast extract concentration, reaching its peak at a concentration of 20 g/L. This suggests that when the yeast extract concentration reached 20 g/L, it approached saturation, and further increasing the nitrogen source had limited effect on promoting strain growth. The effect of different yeast extract concentrations on fermentation was more significant. Protein yield continuously increased with increasing yeast extract concentration, reaching its peak at 20 g/L, while the 25 g/L concentration showed essentially the same level as the 20 g/L concentration. This trend indicates that appropriately increasing the nitrogen source concentration can effectively promote protein synthesis. Protein content showed the same trend as protein yield. This indicates that a high concentration of nitrogen source not only promoted cell growth but also significantly enhanced the protein synthesis capacity of the strain.

#### 3.3.3. Inorganic Salt Optimization

**Figure 4:**
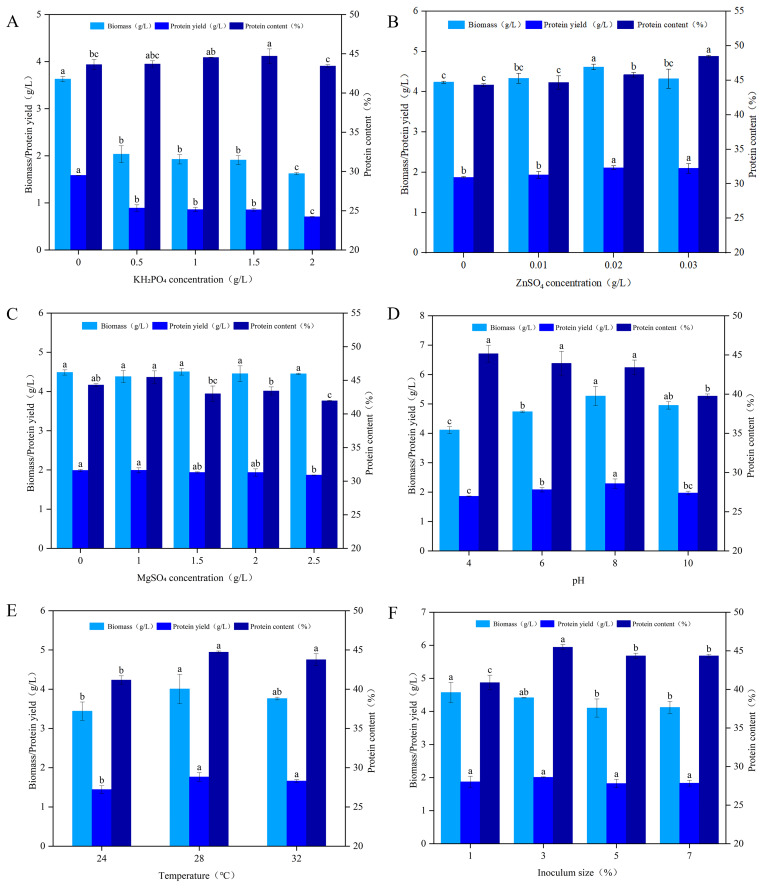
Results of inorganic salt and condition optimization. (**A**) Different concentrations of KH_2_PO_4_. (**B**) Different concentrations of zinc sulfate. (**C**) Different concentrations of MgSO_4_. (**D**) Different pH. (**E**) Different temperatures. (**F**) Different inoculum sizes. Bars bearing different lowercase letters (e.g., a, b, c) differ significantly (*p* < 0.05), whereas bars with the same letter do not. Data are presented as mean ± SD (*n* = 3).

This study investigated the effect of different KH_2_PO_4_ concentrations on Gg6 fermentation. The results are shown in Figure 4A. The experimental results showed that the addition of KH_2_PO_4_ had an inhibitory effect on both cell growth and protein yield. As the KH_2_PO_4_ concentration increased, biomass showed a continuous downward trend, reaching a minimum of 1.6 g/L at 2.0 g/L. This result indicates that under these experimental conditions, the addition of KH_2_PO_4_ was detrimental to strain growth. The addition of KH_2_PO_4_ had no effect on the protein content of the strain, which showed a slow upward trend with increasing KH_2_PO_4_ concentration, indicating that although the addition of KH_2_PO_4_ inhibited cell growth, it did not affect the protein content of the strain. Consequently, protein yield exhibited the same trend as biomass. When no KH_2_PO_4_ was added, protein yield was the highest; as the KH_2_PO_4_ concentration increased, protein yield decreased sharply. This trend further confirms that the addition of KH_2_PO_4_ had a negative impact on strain fermentation. Based on the above results, there is no need to supplement the basal medium with additional inorganic salt ion KH_2_PO_4_.

The effect of ZnSO_4_ concentration on Gg6 fermentation is shown in Figure 4B. The experimental results indicated that ZnSO_4_ concentration had a certain impact on both strain growth and protein yield. As the ZnSO_4_ concentration increased, biomass showed a slow upward trend. When the concentration was 0.02 g/L, biomass reached its maximum value; when the concentration was further increased to 0.03 g/L, biomass decreased slightly, remaining essentially the same as the 0.01 g/L concentration. This indicates that an appropriate amount of ZnSO_4_ had a slight promoting effect on cell growth, but the effect was limited. The addition of ZnSO_4_ had a certain promoting effect on protein synthesis, showing a stable positive effect although the increase was modest. The protein content of the strain showed a continuous upward trend with increasing ZnSO_4_ concentration. These results indicate that the addition of ZnSO_4_ had a positive effect on Gg6 fermentation, mainly reflected in the improvement of protein content, suggesting that an appropriate amount of ZnSO_4_ is beneficial for enhancing protein synthesis.

As shown in Figure 4C, compared with the original basal fermentation medium, the addition of the inorganic salt ion MgSO_4_ had no effect on either the growth or protein synthesis of the Gg6 strain.

#### 3.3.4. Condition Optimization

Culture temperature, inoculum size, and pH are key factors affecting microbial growth and metabolite accumulation. As shown in Figure 4D, pH had a significant effect on the growth of strain Gg6. The biomass reached its maximum at pH 8, indicating that either overly acidic or overly alkaline environments inhibited cell growth to varying degrees. Protein content also varied under different pH conditions. Although protein content showed a decreasing trend as pH increased from 4 to 10, there were no significant differences in protein content among pH 4, 6, and 8. The trend in protein yield was consistent with that of biomass. Protein yield reached its maximum value of 2.3 g/L at pH 8.0, indicating that pH 8.0 was favorable for both cell growth and protein accumulation. Based on the above results, pH has an important effect on the growth and protein synthesis of Gg6. Although protein content was highest at pH 6.0, pH 8.0 was optimal from the perspective of the core indicator of total protein yield.

This study investigated the effects of different culture temperatures (24 °C, 28 °C, 32 °C) on Gg6 fermentation. The results are shown in Figure 4E. The experimental results indicated that culture temperature affected both cell growth and protein synthesis of Gg6. At 28 °C, the biomass, protein content, and protein yield were all higher than those at the other two temperatures. This result indicates that the optimal growth temperature for Gg6 is 28 °C, and temperatures that are too high or too low are unfavorable for effective cell proliferation and protein accumulation. Therefore, 28°C was selected as the fermentation temperature for strain Gg6. As shown in Figure 4F, increasing the inoculum size had no significant effect on protein yield.

#### 3.3.5. Orthogonal and Validation Experiment Results

Based on the single-factor experiment results, an orthogonal experiment was conducted. Four variables that significantly affected the fermentation of strain Gg6 were selected: glucose concentration (A), yeast extract concentration (B), ZnSO_4_ concentration (C), and pH (D). Using protein yield as the evaluation criterion, the orthogonal experiment was designed using SPSS 27. The results are shown in Table 3.

Based on the data analysis in Table 3, the order of influence of the four factors—glucose concentration (A), yeast extract concentration (B), ZnSO_4_ concentration (C), and pH (D)—on protein yield was A > C > D > B, i.e., glucose concentration > ZnSO_4_ concentration > pH > yeast extract concentration. Based on the consideration of protein yield, the optimal combination was determined to be A_2_B_2_C_2_D_1_, namely glucose concentration of 40 g/L, yeast extract concentration of 20 g/L, ZnSO_4_ concentration of 0.02 g/L, and pH of 6. The results of the analysis of variance (ANOVA) are shown in Table 4.

Under the selected optimal conditions, three repeated experiments were conducted. The mean biomass obtained was 11.22 ± 0.23 g/L, the mean protein content was 54.56 ± 0.05%, and the mean protein yield was 6.12 ± 0.11 g/L. Therefore, this optimal condition determined by the orthogonal experiment is considered valid.

### 3.4. Transcriptomic Analysis Based on KH_2_PO_4_ Metabolism

#### 3.4.1. Ion Effect Results

**Figure 5:**
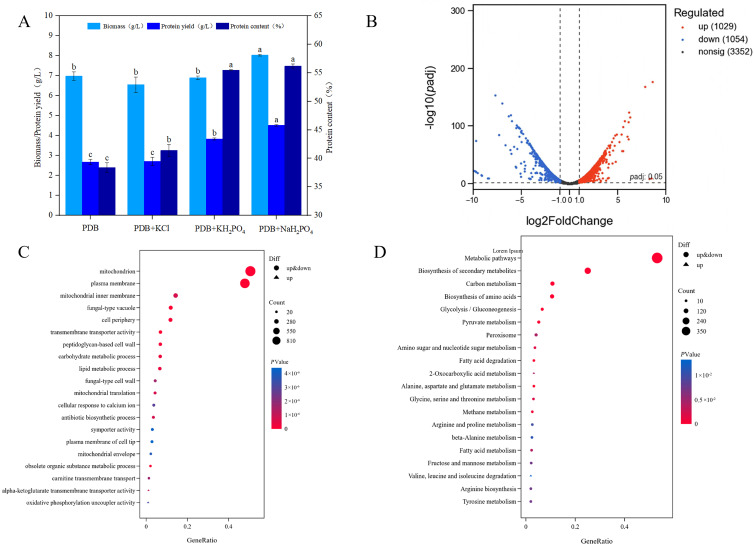
Transcriptome sequencing results. (**A**) Results of ion effect. (**B**) Volcano plot of differentially expressed genes. (**C**) GO annotation analysis. (**D**) KEGG pathway enrichment analysis. Bars bearing different lowercase letters (e.g., a, b, c) differ significantly (*p* < 0.05), whereas bars with the same letter do not. Data are presented as mean ± SD (*n* = 3).

As shown in Figure 5A, after the addition of KCl, there were no significant differences in biomass and protein yield compared with the control group (*p* > 0.05), indicating that K^+^ had no significant promoting effect on the growth and protein synthesis of strain Gg6. After the addition of KH_2_PO_4_, both protein yield and protein content were increased compared with the control group, with significant differences (*p* < 0.05), indicating that KH_2_PO_4_ significantly promoted protein synthesis. After the addition of NaH_2_PO_4_, biomass, protein yield, and protein content were all significantly increased (*p* < 0.05). In summary, phosphate (PO_4_^3−^) is the key ion promoting protein synthesis in *G*. *geotrichum* 6.

#### 3.4.2. Results of 18 Free Amino Acids

As shown in Table 5, in the PDB medium, arginine accounted for 9.63% of the total free amino acids. After KH_2_PO_4_ supplementation, the arginine content significantly increased to 29.48%, representing an approximately 2.2-fold increase, making it the most abundant amino acid. Meanwhile, the glutamic acid content also increased markedly from 8.10% to 16.61%. In contrast, the relative contents of amino acids such as leucine, isoleucine, valine, alanine, proline, and tyrosine decreased significantly. The changes in lysine, threonine, serine, and glycine were relatively minor.

#### 3.4.3. Transcriptome Sequencing Data Analysis

Differentially expressed genes between different treatment samples were screened. Based on the comparison of transcriptome sequencing data, the results are shown in Figure 5B. Under the two conditions (with and without KH_2_PO_4_ addition), a total of 5435 genes were detected. Among them, 1029 genes were up-regulated, while 1054 genes were down-regulated.

Figure 5C shows the GO functional enrichment analysis of differentially expressed genes. The differentially expressed genes were mainly enriched in mitochondria and their inner membrane systems, as well as metabolic processes (carbohydrate metabolic process, lipid metabolism), etc. In terms of molecular function, they were mainly enriched in transmembrane transporter activity and oxidative phosphorylation uncoupling activity. To clarify the metabolic pathways involved in the DEGs, KEGG pathway enrichment analysis was performed.

The analysis results are shown in Figure 5D. Metabolic pathways were the most significantly enriched pathway, followed by the biosynthesis of secondary metabolites, indicating that the strain underwent extensive metabolic pathway regulation and carbon flux redistribution after the addition of KH_2_PO_4_. Pathways related to central carbon metabolism were highly enriched, including carbon metabolism, amino acid biosynthesis, glycolysis, and pyruvate metabolism. These pathways provide sufficient carbon skeletons and energy for amino acid synthesis. Pathways associated with specific amino acid families were also significantly enriched, including the arginine biosynthesis pathway and the glutamate metabolism pathway.

#### 3.4.4. Analysis of Differentially Expressed Genes

The enriched KEGG pathways and expression of differentially expressed genes are summarized in Table 6. Inorganic phosphorus (Pi) is an essential component of various biomolecules responsible for cellular structure and energy storage [23]. Following phosphate treatment, analysis of transcriptomic differential gene expression data revealed that multiple key enzymes in glycolysis—including 6-phosphofructokinase, glyceraldehyde-3-phosphate dehydrogenase, pyruvate kinase (PK), phosphoglycerate kinase (PGK), and enolase—were significantly down-regulated at the gene level. These enzymes perform critical catalytic functions in the glycolytic pathway: 6-phosphofructokinase catalyzes the conversion of fructose-6-phosphate to fructose-1,6-bisphosphate, representing the major rate-limiting step of glycolysis [24]; glyceraldehyde-3-phosphate dehydrogenase catalyzes the oxidation of glyceraldehyde-3-phosphate to 1,3-bisphosphoglycerate while reducing NAD^+^ to NADH [25]; enolase catalyzes the dehydration of 2-phosphoglycerate to phosphoenolpyruvate (PEP); phosphoglycerate kinase (PGK) catalyzes the transfer of the high-energy phosphate group from 1,3-bisphosphoglycerate to ADP, generating ATP and 3-phosphoglycerate, constituting the first substrate-level phosphorylation step in glycolysis [26]; pyruvate kinase (PK) catalyzes the transfer of the high-energy phosphate group from PEP to ADP, producing pyruvate and ATP, representing the final substrate-level phosphorylation stage of glycolysis [27]. The coordinated down-regulation of genes encoding these enzymes not only indicates a significant suppression of overall glycolytic pathway activity but also conveys an important metabolic signal: cells are no longer relying on substrate-level phosphate group transfer as the primary energy production mode, and the flow of phosphate groups is shifting from “substrate-level phosphorylation” toward the more efficient “oxidative phosphorylation” pathway. The overall activity of the glycolytic pathway is significantly inhibited, and the cellular dependence on substrate-level phosphorylation is reduced.

This transition is consistent with the up-regulation of multiple key enzyme-encoding genes in the tricarboxylic acid (TCA) cycle [28,29]. Transcriptomic analysis revealed that the transcriptional levels of citrate synthase (CS), aconitate hydratase, isocitrate dehydrogenase, succinate dehydrogenase flavoprotein subunit, and fumarase were significantly up-regulated, whereas the expression of the 2-oxoglutarate dehydrogenase complex, succinyl-CoA synthetase, and malate dehydrogenase showed no significant changes. Among these, citrate synthase (CS), which catalyzes the condensation of acetyl-CoA and oxaloacetate to form citrate, is the rate-limiting enzyme of the TCA cycle [30]; its up-regulation indicates that more acetyl-CoA enters the TCA cycle [31]. Succinate dehydrogenase (SDH), as mitochondrial electron transport chain complex II, catalyzes the oxidation of succinate to fumarate while directly injecting electrons carried by FADH_2_ into the respiratory chain, serving as a key link between substrate oxidation and phosphorylative energy production [32,33]. The up-regulation of SDH enhances electron injection into the respiratory chain; electrons are transferred through complexes III and IV, driving proton transmembrane transport and ultimately generating proton motive force, which drives ATP synthase to synthesize ATP from ADP and inorganic phosphate (Pi) [34,35]. The significant up-regulation of ATP synthase-encoding genes in the transcriptome further confirms the conclusion that oxidative phosphorylation energy production is enhanced. These results indicate that phosphate treatment significantly increases the cellular capacity for ATP synthesis, providing sufficient energy supply for a series of energy-consuming processes such as protein synthesis and cell growth.

**Figure 6:**
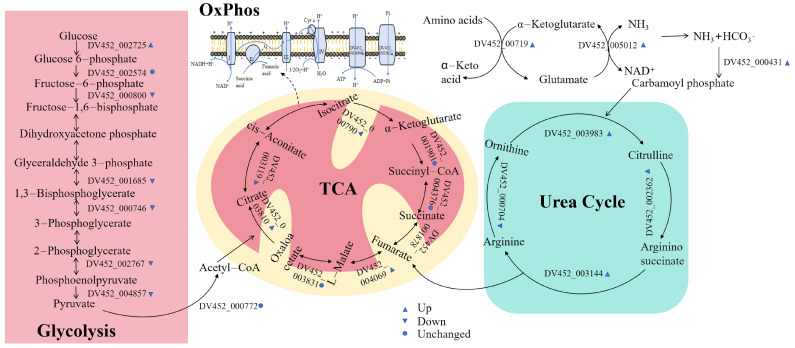
Relevant Metabolic Pathways and Expression of Differentially Expressed Genes. Transcriptional responses under the tested condition are indicated by symbols: ▲, significantly upregulated; ▼, significantly downregulated; ●, no significant differential expression. Black solid arrows denote the direction of metabolic flux, and dashed arrows represent reactions occurring on the inner mitochondrial membrane.

At the level of amino acid metabolism, in the arginine biosynthesis pathway, the up-regulation of genes encoding carbamoyl phosphate synthase, ornithine aminomethyltransferase, argininosuccinate synthase, and argininosuccinate lyase, together with the down-regulation of the arginase-encoding gene, collectively achieve net arginine accumulation [36,37,38,39,40]. This expression pattern provides a molecular explanation for the significantly increased arginine content observed in the amino acid analysis. The mechanism is illustrated in Figure 6.

## 4. Discussion and Conclusions

In this study, a strain of *Geotrichum candidum* (designated as *Geotrichum candidum* 6) was successfully isolated from cheese. Through single-factor experiments and orthogonal array design for shake flask fermentation optimization, the protein yield reached 6.12 g/L, which is close to the high-yield levels previously reported in the literature [41], indicating that *G. candidum* 6 has potential as a single-cell protein production strain. In investigating the regulatory mechanism by which the inorganic salt KH_2_PO_4_ enhances protein yield, ion effect analysis revealed that the promoting effect of KH_2_PO_4_ is mainly attributed to the phosphate ion (PO_4_^3−^) rather than K^+^. Amino acid analysis showed that upon KH_2_PO_4_ supplementation, the relative content of arginine increased from 9.63% to 29.48% (an approximately 2.2-fold increase), and glutamate increased from 8.10% to 16.61%.

Transcriptomic analysis further elucidated the molecular mechanism. At the carbon metabolism level, following KH_2_PO_4_ treatment, key glycolytic enzyme genes including 6-phosphofructokinase, glyceraldehyde-3-phosphate dehydrogenase, phosphoglycerate kinase, enolase, and pyruvate kinase were significantly downregulated, while key TCA cycle enzyme genes including citrate synthase, aconitate hydratase, isocitrate dehydrogenase, succinate dehydrogenase, and fumarase were significantly upregulated. This expression pattern indicates that phosphate redirects carbon flux from glycolysis to the TCA cycle. Similarly, Chung et al. [42] observed that phosphate alters carbon flux distribution and promotes glycogen and protein accumulation in halophilic cyanobacteria, which is consistent with our findings. Regarding the association between phosphate and energy metabolism, ATP synthase-encoding genes were significantly upregulated, suggesting enhanced oxidative phosphorylation capacity. Chouayekh and Virolle [43] demonstrated that in *Streptomyces* lividans, polyphosphate kinase can both catalyze ATP synthesis from polyphosphate and regenerate ATP from polyphosphate, revealing a direct coupling mechanism between phosphate metabolism and energy metabolism. This finding aligns with the significant upregulation of ATP synthase-encoding genes in our study, collectively supporting the conclusion that phosphate enhances oxidative phosphorylation for energy production. At the nitrogen metabolism level, four key enzyme genes in the arginine biosynthesis pathway (*cpa*, *arg3*, *arg1*, *arg4*) were significantly upregulated, while the arginase-encoding gene (*car1*) was downregulated, which forms the molecular basis for arginine accumulation. Glutamate dehydrogenase (*gdh*) was significantly upregulated, consistent with the evidence reported by Rodríguez-García et al. [44] that phosphate signaling regulates nitrogen metabolism.

Notably, KH_2_PO_4_ promoted protein synthesis in the low-nitrogen medium (5% nitrogen content) but exhibited an inhibitory effect in the high-nitrogen medium (18% nitrogen content). This difference arises from the distinct carbon–nitrogen–phosphorus (C:N:P) stoichiometric backgrounds of the two culture media. Under the low-nitrogen background, nitrogen limitation triggers the downregulation of ribosomal protein genes in filamentous fungi, leading to active suppression of protein synthesis [45]. Supplementation with KH_2_PO_4_ provides phosphorus, which is essential for ribosomal RNA synthesis, and may relieve this suppression, thereby promoting protein synthesis. Under the high-nitrogen background, the high concentration of nitrogen sources may impose osmotic or ionic stress on the fungus, and the additional KH_2_PO_4_ could further exacerbate this stress.

In addition to the above mechanistic insights, some methodological limitations of this study should also be acknowledged. For example, response surface methodology is more capable than orthogonal design in capturing interactions among variables, but this method was not employed in the present study. Furthermore, all experiments were conducted at the shake-flask scale, and the industrial applicability of the optimized conditions remains to be validated in a controlled bioreactor.

In summary, this study systematically elucidates the potential molecular mechanism by which phosphate ion (PO_4_^3−^) promotes protein synthesis in *Geotrichum candidum* 6 through carbon metabolic reprogramming and synergistic regulation of arginine metabolism, and confirms that *G. candidum* 6 has high-yield potential for microbial protein production, providing a practical basis for its application in feed protein production. The transcriptomic data point to potential targets for further metabolic engineering. Future studies should employ response surface methodology to further optimize culture conditions, conduct scale-up validation in controlled bioreactors, measure osmotic pressure and compatible solutes to test the osmotic stress hypothesis under high-nitrogen conditions, and explore alternative agricultural waste substrates as well as animal feeding trials to assess practical applicability and safety.

## Figures and Tables

**Table 1:** Factors and levels of orthogonal test.

Factors	Level 1	Level 2	Level 3
glucose concentration (A) g/L	20	40	60
yeast extract powder concentration (B) g/L	15	20	25
ZnSO_4_ concentration (C) g/L	0.01	0.02	0.03
pH (D)	6	8	10

**Table 2:** Orthogonal test design.

n	Glucose Concentration (g/L)	Yeast Extract Powder Concentration (g/L)	ZnSO_4_ Concentration (g/L)	pH
1	20	15	0.01	6
2	20	20	0.02	8
3	20	25	0.03	10
4	40	15	0.02	10
5	40	20	0.03	6
6	40	25	0.01	8
7	60	15	0.03	8
8	60	20	0.01	10
9	60	25	0.02	6

**Table 3:** Orthogonal experimental design and results.

Test No.	A Glucose Concentration (g/L)	B Yeast Extract Concentration (g/L)	C ZnSO_4_ Concentration (g/L)	D pH	Protein Yield (g/L)
1	1	1	0.01	1	4.34 ± 0.01
2	1	2	0.02	2	4.87 ± 0.03
3	1	3	0.03	3	4.74 ± 0.06
4	2	1	0.02	3	6.09 ± 0.01
5	2	2	0.03	1	6.03 ± 0.05
6	2	3	0.01	2	5.33 ± 0.00
7	3	1	0.03	2	4.60 ± 0.04
8	3	2	0.01	3	4.72 ± 0.11
9	3	3	0.02	1	5.39 ± 0.06
K1	13.939	15.028	14.389	15.760	
K2	17.449	15.620	16.346	14.800	
K3	14.720	15.460	15.373	15.548	
k1	4.646	5.009	4.796	5.253	
k2	5.816	5.207	5.449	4.933	
k3	4.907	5.153	5.124	5.183	
R	1.170	0.198	0.652	0.320	

Note: All values are presented as mean ± SD. SD values were rounded to two decimal places for consistency with the mean values.

**Table 4:** Analysis of variance (ANOVA) for orthogonal experiment.

Factor	Sum of Squares	df	Mean Square	F-Value	Significance
A Glucose concentration (g/L)	6.793	2	3.397	1240.850	**
B Yeast extract concentration (g/L)	0.188	2	0.094	34.304	**
C ZnSO_4_ concentration (mg/L)	1.915	2	0.958	349.807	**
D pH	0.508	2	0.254	92.881	**
Error	0.0494	18	0.003		
Total	9.4537	26			

Note: ** indicates an extremely significant difference (*p* < 0.01).

**Table 5:** Effects of KH_2_PO_4_ on the free amino acid content.

Amino Acid Category	Amino Acid	PDB (%)	PDB + KH_2_PO_4_ (%)
	Lysine	9.49	10.39
	Threonine	11.04	10.78
	Methionine	1.47	0.28
Essential amino acids	Phenylalanine	3.46	1.54
	Leucine	5.23	1.89
	Isoleucine	3.87	1.60
	Valine	6.67	2.02
	Histidine	6.83	4.77
	Tryptophan	0.72	0.65
	Glutamic acid	8.10	16.61
	Aspartic acid	3.00	1.56
	Serine	10.91	8.20
	Alanine	9.69	4.65
Non-essential amino acids	Glycine	2.12	2.08
	Proline	3.32	1.41
	Arginine	9.63	29.48
	Tyrosine	2.77	0.84
	Cystine	1.68	1.25
Total amino acid content	-	100	100

**Table 6:** Enriched pathways and expression of differentially expressed genes.

KEGG Pathway	KEGG Ortholog (KO Name)	KO ID	Gene ID	log2FC	*p*adj	Gene Expression
Glycolysis	Hexokinase 1	K00844	DV452_002725	1.31	1.93 × 10^−9^	Up
	Glucose-6-phosphate isomerase	K01810	DV452_002574	−0.46	3.79 × 10^−2^	Not significant
	6-Phosphofructokinase	K00850	DV452_000800	−2.01	2.74 × 10^−20^	Down
	Glyceraldehyde-3-phosphate dehydrogenase	K00134	DV452_001685	−3.16	2.15 × 10^−44^	Down
	Phosphoglycerate kinase	K00927	DV452_000746	−2.87	1.28 × 10^−37^	Down
	Enolase	K01689	DV452_002767	−3.67	9.04 × 10^−57^	Down
	Pyruvate kinase	K00873	DV452_004857	−2.90	2.42 × 10^−38^	Down
Oxidative phosphorylation	ATP synthase	K02133	DV452_003699	1.24	7.49 × 10^−6^	Up
	Phosphate transporter	K15102	DV452_000382	1.07	6.22 × 10^−7^	Up
Pyruvate metabolism	Pyruvate dehydrogenase E1 component subunit beta	K00162	DV452_000772	0.16	0.50	Not significant
TCA	Citrate synthase	K01647	DV452_003810	1.71	2.31 × 10^−15^	Up
	Aconitate hydratase	K01681	DV452_003119	1.40	9.21 × 10^−11^	Up
	Isocitrate dehydrogenase	K00031	DV452_000790	3.68	5.85 × 10^−57^	Up
	2-Oxoglutarate dehydrogenase complex	K00658	DV452_001901	0.23	0.30	Not significant
	Succinyl-CoA synthetase	K01900	DV452_004376	−0.55	0.01	Not significant
	Succinate dehydrogenase flavoprotein subunit	K00235	DV452_001878	1.93	7.02 × 10^−19^	Up
	Fumarase	K01679	DV452_004069	1.37	2.82 × 10^−10^	Up
	Malate dehydrogenase	K00026	DV452_003831	0.82	1.37 × 10^−4^	Not significant
Alanine, aspartate and glutamate metabolism	Alanine aminotransferase	K00814	DV452_000719	4.85	5.62 × 10^−86^	Up
	NADP-specific glutamate dehydrogenase	K00262	DV452_005012	2.62	3.56 × 10^−32^	Up
	Carbamoyl phosphate synthase	K01955	DV452_000431	1.75	1.00 × 10^−15^	Up
Arginine biosynthesis	Ornithine aminomethyltransferase	K00611	DV452_003983	2.98	1.33 × 10^−37^	Up
	Argininosuccinate synthase	K01940	DV452_002362	1.83	3.26 × 10^−17^	Up
	Argininosuccinate lyase	K01755	DV452_003144	2.28	6.27 × 10^−24^	Up
	Arginase	K01476	DV452_000704	−2.50	1.17 × 10^−27^	Down

Note: “Not significant” indicates that the gene did not meet the significance threshold (|log_2_FC| < 1 or *p*adj > 0.05).

## Data Availability

The original contributions presented in this study are included in the article. Further inquiries can be directed to the corresponding authors.

## Abbreviations

The following abbreviations are used in this manuscript:

SCP	Single-cell protein
MPs	Microbial proteins
ITS	Internal transcribed spacer
PCR	Polymerase chain reaction
NCBI	National Center for Biotechnology Information
PDB	Potato Dextrose Broth
PDA	Potato Dextrose Agar
DNA	Deoxyribonucleic acid
RNA	Ribonucleic acid
TCA	Tricarboxylic acid
ATP	Adenosine triphosphate
ADP	Adenosine diphosphate
NAD^+^	Nicotinamide adenine dinucleotide (oxidized)
NADH	Nicotinamide adenine dinucleotide (reduced)
FADH_2_	Flavin adenine dinucleotide (reduced)
PEP	Phosphoenolpyruvate
PK	Pyruvate kinase
PGK	Phosphoglycerate kinase
CS	Citrate synthase
SDH	Succinate dehydrogenase
GO	Gene Ontology
KEGG	Kyoto Encyclopedia of Genes and Genomes
DEGs	Differentially expressed genes
ANOVA	Analysis of variance
SPSS	Statistical Package for the Social Sciences
SD	Standard deviation
